# Identity, Acculturation, and E-Cigarette Use among Bicultural Hispanic Youth and Young Adults

**DOI:** 10.1089/heq.2024.0013

**Published:** 2024-08-19

**Authors:** Emily C. Sanders, Sarah Evans, Alex Budenz, N. Yvette Frias, Sarah Byrnes, Everly Macario

**Affiliations:** ^1^ Food and Drug Administration, Center for Tobacco Products, Silver Spring, Maryland, USA.; ^2^ IQ Solutions, North Bethesda, Maryland, USA.

**Keywords:** Hispanic, smoking prevention, e-cigarettes, youth, health disparities, young adults

## Abstract

**Introduction:**

Research suggests Hispanic/Latino/a/x/e (hereafter Hispanic) youth/young adult (YYA) tobacco use may vary by acculturation level, but few studies have explored e-cigarette use by acculturation or how bicultural identity may affect tobacco susceptibility. This study examined acculturation’s role in U.S. Hispanic YYA e-cigarette use to better understand risk and protective factors.

**Methods:**

We conducted 20 virtual focus groups in English/Spanish with Hispanic 13–24-year-olds (December 2021–January 2022). We coded transcripts in their original language (intercoder reliability kappa 0.89) and conducted thematic analysis segmented by age cohort, e-cigarette use, and acculturation level.

**Results:**

More acculturated participants had greater knowledge/familiarity with tobacco/nicotine compared with less acculturated participants. Bicultural participants more commonly mentioned curiosity and direct peer pressure as e-cigarette use drivers. While bicultural participants noted the negative impacts of e-cigarette use on family relationships, this was not a use deterrent. Less acculturated participants were most concerned with stigma, negative family impacts, and broader Hispanic community disapproval.

**Discussion:**

This study suggests that differences related to Hispanic identity and the acculturative process may increase or decrease e-cigarette use risk. Bicultural YYA, who represent more than half of U.S. Hispanic YYA, toggle between Hispanic roots and mainstream U.S. culture, which can lead to unique stressors that may increase susceptibility to e-cigarettes.

**Health Equity Implications:**

Public health efforts must recognize the heterogeneity of the Hispanic population and the role acculturation plays in e-cigarette use. A nuanced understanding can inform the design of targeted and effective public health strategies to reduce disparities in e-cigarette risk and use.

## Introduction

U.S. Hispanic and Latino/a/x/e (hereafter Hispanic^1^) youth (ages 12–17) and young adults (ages 18–25) (hereafter YYA) face morbidity and mortality risks related to tobacco use.^1^ Approximately one-quarter (23.8%) of Hispanic middle and high school youth in 2023 had ever used any tobacco product, and at least 1 in 10 (11.7%) had used within the past 30 days.^1^ Among Hispanic young adults in 2021, 42.1% had ever used any tobacco product, and 15.9% used within the past 30 days.^2^

Over the last decade, electronic nicotine delivery systems (hereafter e-cigarettes) have become the most commonly used tobacco product among U.S. YYA.^3^ Among Hispanic youth in 2023, 18.2% had ever used e-cigarettes, and 8.5% used within the past 30 days.^1^ Among Hispanic young adults in 2021, 19.8% had ever used e-cigarettes, and 7.9% reported past 30-day use.^2^

Previous research suggests that Hispanic youth have high levels of curiosity about and may initiate tobacco use with e-cigarettes.^4^ Data from the 2022 National Youth Tobacco Survey show that Hispanic youth (12–17) are susceptible to e-cigarettes (35.7%) and curious about them (35.6%).^5^ This may partly be attributed to Hispanic youth perceiving e-cigarettes as less harmful than cigarettes (45.2%) or believing that they do not know enough to evaluate the relative harms (29.2%) or addictiveness (41.0%) of e-cigarettes compared with cigarettes.^6^

The Hispanic population is the largest racial or ethnic minority population in the United States (19%)^7^ and is highly diverse. Research shows that risk and protective factors for tobacco use may differ by Hispanic subgroup.^8,9^ One factor that may play an important role in e-cigarette use is acculturation, the complex process of blending into the mainstream culture while navigating one’s heritage culture.^10^ Prior research has shown that Hispanic YYA may face differential risks of combustible tobacco use due to stress, internal conflict, or intrafamilial conflict that may be experienced as part of the acculturation process.^11,12^ In some cases, acculturating to the U.S. mainstream is associated with worse health behaviors and outcomes compared with those adhering to the heritage culture among both Hispanic adults^13,14^ and youth.^15^ Research shows that higher acculturation level,^13,16,17^ higher acculturative stress,^11^ U.S.-born nativity status,^18^ having nonimmigrant parents,^19^ having U.S.-oriented friends,^20^ and English language preference^21–24^ are positively associated with combustible tobacco use among Hispanic people.

Few studies have explored acculturation and e-cigarette use among Hispanic YYA.^25^ Even less is understood about how the unique experiences of bicultural Hispanic YYA, who represent more than half of U.S. Hispanic YYA,^26^ may affect tobacco use risk. Being “bicultural” signifies the embrace of two cultures simultaneously. Bicultural Hispanic YYAs can feel culturally connected to both their U.S. and Hispanic identities.^27^ This is a salient area of inquiry, as more than 9 in 10 Hispanic children living in the United States were born in the United States, but over half have at least one foreign-born parent.^28^ For Hispanic YYA, pressure to conform to U.S. culture may conflict with parents’ heritage culture and contribute to acculturative stress.

This study sought to better understand risk and protective factors for e-cigarette use among Hispanic YYA and explore variations by acculturation level, with a specific focus on understanding the experiences of bicultural YYA.

## Methodology

In December 2021–January 2022, we conducted 20 (16 English, 4 Spanish) virtual focus groups (90 minutes each in duration) with 95 Hispanic YYA ages 13–24 who were not closed to using e-cigarettes and were not established users of e-cigarettes (approved by Salus IRB, no. 22095-01). For this study, we included participants who self-identified as having Hispanic ethnicity. We operationalized “Hispanic” as having heritage from a Spanish-speaking county (i.e., South America, Central America, the Caribbean, and Spain) given the association between linguistic acculturation and combustible tobacco use.^21–24^ Our focus was understanding the experiences of YYA who either immigrated to the United States themselves or their families had immigrated to the United States.

Because this study was conducted during the COVID-19 pandemic, we conducted all focus groups online. We first segmented groups by age (13–14, 15–17, and 18–24 years). Within age-groups, we segmented by acculturation level, which was measured by language preference and birthplace^29^ (Table 1). While many measures of acculturation are available in the literature, we selected these based on precedence in the tobacco control literature^31,32^ and consultation with subject matter experts. Within age by acculturation combinations, we segmented by e-cigarette use status (susceptible/nontriers, recent but not frequent users, and every day/frequent current users; Table 1). We excluded participants reporting more than 100 lifetime cigarettes from the study as a proxy measure for established tobacco use. Population prevalence and research goals led to some segment combinations being represented by more than one group (e.g., 18–24, more acculturated, frequent use), whereas some segment combinations were not represented (e.g., 13–14, less acculturated). The full segmentation is presented in Table 2.

**Table 1.** Segment Definitions

Segmentation characteristic	Definition
Acculturation level	
Higher acculturation	Answered “Only English” or “Mostly English” to the question: *What language(s) do you usually speak with your family?* Coded answer was “U.S.-born” to the question: *Where were you born?*
Lower acculturation	Answered “Only Spanish” or “Mostly Spanish” to the question: *What language(s) do you usually speak with your family?* Coded answer was “born outside the U.S.” to the question: *Where were you born?*
Bicultural	Answered “Only English” or “Mostly English” to the question: *What language(s) do you usually speak with your family?* AND coded answer was “born outside the U.S.” to the question: *Where were you born?* OR Answered “Only Spanish” or “Mostly Spanish” to the question: *What language(s) do you usually speak with your family?* AND coded answer was “U.S.-born” to the question: *Where were you born?* OR Answered “Spanish or English equally” to the question: *What language(s) do you usually speak with your family?* AND coded answer was “U.S.-born” or “born outside the U.S.” to the question: *Where were you born?*
E-cigarette use status	
Susceptible/non-triers	Answered “no” to the question: *Have you ever tried using an e-cigarette, vape pen, mod, or disposable device (like Puff Bar, Suorin, Vuse, Blu, JUUL, Logic, NJoy, or SMOK), even one or two puffs?* Susceptibility measured by indicating anything other than “definitely not” to the following questions: *“Do you think you will use an e-cigarette, vape pen, mod, or disposable device (like Puff Bar, Suorin, Vuse, Blu, JUUL, Logic, NJoy, or SMOK) in the next year?”; “Do you think you will use an e-cigarette, vape pen, mod, or disposable device (like Puff Bar, Suorin, Vuse, Blu, JUUL, Logic, NJoy, or SMOK) soon?”; and “If one of your best friends were to offer you an e-cigarette, vape pen, mod, or disposable device (like Puff Bar, Suorin, Vuse, Blu, JUUL, Logic, NJoy, or SMOK), would you use it?”* Response options ranged from “definitely not” to “definitely yes” (based on an adapted adolescent susceptibility to smoking scale^30^).
Recent but not frequent users	Answered “yes” to the question: *Have you ever tried using an e-cigarette, vape pen, mod, or disposable device (like Puff Bar, Suorin, Vuse, Blu, JUUL, Logic, NJoy, or SMOK), even one or two puffs?* Reports e-cigarette use “some days” or “rarely” to the question: *During the past 30 days, how often did you use an e-cigarette, vape pen, mod, or disposable device (like Puff Bar, Suorin, Vuse, Blu, JUUL, Logic, NJoy, or SMOK)? Would you say.. .”* Answer choices are every day, most days, some days, rarely, and not at all.
Every day/frequent current users	Answered “yes” to the question: *Have you ever tried using an e-cigarette, vape pen, mod, or disposable device (like Puff Bar, Suorin, Vuse, Blu, JUUL, Logic, NJoy, or SMOK), even one or two puffs?* Answered “most days” or “every day” in the past 30 days to the question: *During the past 30 days, how often did you use an e-cigarette, vape pen, mod, or disposable device (like Puff Bar, Suorin, Vuse, Blu, JUUL, Logic, NJoy, or SMOK)? Would you say.. .”* Answer choices are every day, most days, some days, rarely, and not at all.

**Table 2.** Segmentation Plan: Characteristics of the 20 Focus Groups

	Ages 13–14	Ages 15–17	Ages 18–24		
More acculturated	(1) Recent but not frequent user; English	(5) Recent but not frequent user; English	More acculturated	(11) Recent but not frequent user; English	(17) Frequent/Every day current user; English
	(2) Susceptible nontrier; English	(6) Susceptible nontrier; English		(12) Recent but not frequent user; English	(18) Frequent/Every day current user; English
Less acculturated		(7) Recent but not frequent user; Spanish	Less acculturated	(13) Recent but not frequent user; Spanish	
		(8) Susceptible nontrier; Spanish		(14) Recent but not frequent user; Spanish	
Bicultural	(3) Recent but not frequent user; English	(9) Recent but not frequent user; English	Bicultural	(15) Recent but not frequent user; English	(19) Frequent/Every day current user; English
	(4) Susceptible nontrier; English	(10) Susceptible nontrier; English		(16) Recent but not frequent user; English	(20) Frequent/Every day current user; English

Two professional recruitment firms recruited participants representing a diverse mix of characteristics (e.g., national background, race, and gender) assessed via the screener and demographic survey. These recruitment firms, one of which specializes in the recruitment of Hispanic people, have built databases of potential participants through direct-to-community touchpoints including faith-based organizations. We provided participants with an electronic gift card as a token of appreciation. Parents/guardians provided consent for youth to participate, youth provided assent to participate, and young adults provided consent to participate.

A bilingual/bicultural (of Mexican heritage) moderator facilitated a discussion, which we audio-recorded and transcribed. Native Spanish speakers transcribed the Spanish language documents. The semistructured guide explored Hispanic identity; tobacco/nicotine knowledge and familiarity; e-cigarette knowledge, attitudes, and beliefs; and reasons for e-cigarette use/curiosity.

We conducted a thematic analysis of the data, exploring patterns and themes at the study level and by focus group segment.^33,34^ Our codebook contained deductive (hypothesized themes based on extant literature on tobacco use among Hispanic YYA) and inductive codes (emergent themes grounded in the data) and definitions.^35^ Three coders, including two native Spanish speakers, coded all transcripts in their original language using Dedoose software. Analysts double-coded five transcripts (one to calibrate and refine the codebook and four for paired-coding, representing one-quarter of all transcripts). The final kappa across the four transcripts included in paired-coding showed high intercoder reliability at 0.89.

## Results

### Participant characteristics

Ninety-five Hispanic YYA participated (Table 3). Participants represented a range of racial and national backgrounds (15 Spanish-speaking countries). Segment-based analyses showed few noteworthy differences by age or e-cigarette use status; therefore, we present findings by acculturation level. Unless otherwise specified, the following thematic findings were consistent across participants.

**Table 3.** Participant Characteristics

Segment/demographic characteristic	*n* (%)
Age segment	
13–14	18 (18.9%)
15–17	29 (30.5%)
18–24	48 (50.5%)
E-cigarette use segment	
Frequent	20 (21.1%)
Recent not frequent	55 (57.9%)
Susceptible nontrier	20 (21.1%)
Acculturation segment	
More acculturated	39 (41.1%)
Bicultural	37 (38.9%)
Less acculturated	19 (20.0%)
Race	
American Indian or Alaska Native	2 (2.1%)
Black or African American	20 (21.1%)
Mestizo/a	13 (13.7%)
Native Hawaiian or Other Pacific Islander	1 (1.1%)
White	32 (33.7%)
Other^a^	26 (27.4%)
Refused	1 (1.1%)
Hispanic subgroup	
Mexican, Mexican American, Chicano, or Chicana	33 (34.7%)
Puerto Rican	13 (13.7%)
Cuban	5 (5.3%)
Dominican	11 (11.6%)
Other only^b^	30 (31.6%)
Multiple selected	3 (3.2%)
Gender identity	
Girl/Woman	45 (47.4%)
Boy/Man	48 (50.5%)
Gender queer/gender fluid/nonbinary/gender nonconforming/agender	2 (2.1%)
Total	95 (100%)

^a^ “Other” responses included “brown” (1), Colombian (1), El Salvador (2), Guatemala (2), Hispanic (6), Honduran (2), Mexican (5), Middle Eastern (1), “multi-racial” (1), Salvadoran (2), Spanish (1), Refused (2).

^b^ “Other” responses included Argentina (1), Bolivia (1), Colombia (7), Costa Rica (2), Ecuador (4), El Salvador (2), Guatemala (2), Honduran (3), Panama (1), Peruvian (2), Portuguese/Brazil (1), Salvadoran (3), and Spanish [from Spain] (1).

#### Theme #1: Hispanic heritage is a source of pride but also comes with challenges

Discussions began with explorations of Hispanic identity from images participants selected in advance to represent their ethnicity. Hispanic YYA demonstrated pride in their heritage (Table 4, 1.1). Participants across acculturation segments cited music, dance, and sports as inextricably linked to their culture and creating a sense of unity. More acculturated and bicultural segments discussed these topics more frequently. Participants in the bicultural and lower acculturation segments stressed the importance of family (“familismo”), highlighting attachment, responsibility, and loyalty to families. Bicultural and higher acculturation segments talked about hard work and self-improvement as core values.

**Table 4.** Verbatim Excerpts by Thematic Finding

Thematic finding	Excerpt ID	Verbatim excerpt	Participant segment
Theme #1: Hispanic heritage is a source of pride but also comes with challenges.	1.1	*“I feel like a big part of my journey as a Hispanic, or as a Colombian, is to keep up the traditions I had while growing up… I still cook certain foods that I have cooked all the time that I grew up eating at Christmas time… It’s a way of carrying on and still feeling connected even if I’m not like really around other Hispanic people. It is still what I grew up with. And it’s what I have known. So, it’s—it sounds corny, but it is really a part of me, and I just want to keep on doing it.”*	18–24, Recent not frequent use, bicultural
	1.2	*“It is very common for Hispanic families to have more conservative views, like the older generation. So, it sucks when the newer generation is open-minded on different things—especially when it comes to like, U.S. culture, and being open to more diverse ideology. It’s like that separation makes it kind of difficult at times.”*	18–24, Recent not frequent use, bicultural
	1.3	“Yo diría que es una comunidad fuerte cuando nos identificamos como Latinos. Siempre se sabe que hay confianza entre nosotros. Tenemos experiencias muy similares. Será por lo que sufrimos igual… por la historia que tenemos, hemos transformado este país.” [*“*I would say that it is a strong community when we identify ourselves as Latinos. You always know that there is trust among us. We have very similar experiences. It’s likely because of what we commonly endure … because of the history we have, we have transformed this country*.”*]	18–24, Recent not frequent use, less acculturated
Theme #2: Participants were confused about, and held misperceptions around, the relationship between nicotine and tobacco.	2.1	*“I’m not really sure. I know that tobacco and nicotine are both addictive...I feel like all of these would be on that list [images of eight tobacco products on the shared screen] of stuff to avoid … [but] I wouldn’t really know how to differentiate between either one of them.”*	15–17, Susceptible nontrier, bicultural
	2.2	*“I kind of go off the blind assumption that if you have to roll it, it might contain some tobacco. But if it’s electronic, there might just be nicotine in it.”*	18–24, Recent not frequent, bicultural
Theme #3: E-cigarettes are familiar and popular among Hispanic YYA, with the term “vape” used in English and Spanish.	3.1	*“It’s a consumer thing now… there [are] Asian people vaping, there [are] Hispanics, there [are] White people. A lot of White people use vapes. I think that’s where we got that from. And we kind of like just saw them and it was, ‘Ooh, cool gadget that they are using.’ And then it was like — boom. And we started using it. I don’t really think that it is a race thing.”*	18–24, Frequent, bicultural
Theme #4: Perceptions of e-cigarette harm were polarized.	4.1	*“So, it’s about what you put in it [the vape]. So, you can control the juice that you put in it and how much nicotine content it has, the percentage that it has, if it even has any at all.”*	18–24, Recent not frequent, bicultural
	4.2	*“…because like everybody knows everything about cigarettes are bad. That the actual cigarette is bad. But there is a lot of gray areas surrounding vapes. ‘Oh, well vaping is just like water vapor. So, it’s not bad. I mean, I drink water. So why is vaping bad?’ And then they will try and vape and then they are like, ‘Oh, nothing is happening to me. Like my voice is not raspy, like most smokers are. My lungs are—I can breathe.’ So, they are, like, ‘Let me just stay with vaping.’”*	15–17, Susceptible non-trier, more acculturated
	4.3	*“I feel like the Hispanic people and people of color, in general, like, are the ones that are the most affected. Because we kind of like — I don’t want to say that we have like anything to fall back on, but most White people have like the resources to like get out of the situation where they might be addicted. They have the money to, like, fuel their addiction or like keep getting the resources … Whereas, Hispanic people or people of color might not have the same opportunities. So, addiction can affect them in different ways.”*	15–17, Susceptible nontrier, bicultural
Theme #5: Curiosity drives e-cigarette initiation while continued use is driven by peers and perceived mental health benefits.	5.1	*“They want to try it and see what it feels like. Like, what’s the hype? Sometimes you won’t get why they are doing it every day. It’s like, ‘Why are you doing it every day? Does it even feel good? Like what do you get out of it?’ So, you want to find out what they are feeling, I would say. Just curiosity, in general.”*	18–24, Recent not frequent use, more acculturated
	5.2	*“Personally, I don’t do it, but if [I] saw one of my Hispanic or Latino classmates … [if I] were to see a group of people smoking like a vape, or something, they’d be like, ‘Oh, everyone else is doing it. I should be doing it, too. I can fit in.’ It’s mostly the fitting in part of it.”*	15–17, Recent not frequent, more acculturated
	5.3	* ”I think that various factors come into play. Maybe they use it as a way to de-stress or, like, to deal with stuff. Or sometimes the nicotine levels that the product that they are using has... Maybe it hooks them and their body reacts to it and their brain keeps telling them, ‘Oh, maybe let’s do it again, next time.’ Or, they just get hooked on it.”*	18–24, Frequent, bicultural
	5.4	*“It’s usually just people that are—I don’t know—in my school, it’s different. None of them want to look cool. Sometimes it is just like because of anxiety or they don’t want to deal with something or they’re sad. Most of them don’t really do it to act cool.”*	13–14, Susceptible nontrier, bicultural
Theme #6: Hispanic YYA face unique risk and protective factors based on their acculturation level.	6.1	*“I feel like it is just ingrained in us, to actually move forward, pay it, and contribute back to whoever paved the way for us to be here. So, it is really important for us, as Hispanics—like my parents came here and risked everything for us. So, the least we can do is pay it forward to whoever is next.”*	18–24, Frequent current use, bicultural

Participants also voiced challenges to being Hispanic in the United States. Several participants spoke of disconnect/discord in older versus younger generations’ values (Table 4, 1.2). Participants commonly mentioned views around traditional gender roles and wanting to break traditional molds of *machismo* (being “manly” and self-reliant, and entitlement to dominate^36^) and *marianismo* (the role of women as family- and home-centered, encouraging passivity, self-sacrifice, and chastity^36^). Participants also spoke of discrimination experiences (both observed and direct). This included out-group discrimination (i.e., discrimination from outside the Hispanic population, specifically from White Americans) based on skin color, assumptions about English language proficiency, judgment due to speaking Spanish or having an accent, prejudice in workplaces and schools, and feeling the need to “suppress” their Hispanicity. Several additionally reported feelings of in-group discrimination (i.e., discrimination from within the Hispanic population) due to a lack of Spanish language proficiency, being LGBTQ+, or not conforming to traditional norms.

#### Theme #2: Participants were confused about, and held misperceptions around, the relationship between nicotine and tobacco

Knowledge about tobacco and nicotine varied by acculturation, with bicultural and higher acculturation segments showing greater overall familiarity with these products compared with lower acculturation segments. Participants said they knew if products contained tobacco primarily by the way they look or smell, from tobacco use prevention programs in school focused on combustible products, or labels on tobacco product packaging. Participants were less certain about the relationship between nicotine and tobacco (Table 4, 2.1). Participants were also uncertain whether e-cigarettes are a tobacco product; many considered e-cigarettes to be tobacco-free due to the labeling on e-cigarettes. Most participants believed, however, that e-cigarettes contain nicotine, explaining they knew this by the packaging or “warning labels.”^2^

Across groups, participants correctly linked nicotine to addiction. Participants were divided on the association between nicotine and physical health effects; some held misperceptions that nicotine could cause cancer, though participants more frequently linked combustion (not nicotine) to physical health consequences. Participants frequently stated that nicotine is harmful only after repeated exposure or exposure at high levels (Theme #4). Further, participants associated nicotine with e-cigarettes, and participants more often identified e-cigarettes as “nicotine products” while identifying combustible products as “tobacco products,” for which the relationship with nicotine was less clear (Table 4, 2.2).

#### Theme #3: E-cigarettes are familiar and popular among Hispanic YYA, with the term “vape” used in English and Spanish

Participants reported that e-cigarette use has become common across races and ethnicities (Table 4, 3.1). Participants across acculturation levels were familiar with a range of e-cigarette devices (e.g., pods and cartridges) and brands. Notably, the vocabulary used by participants in talking about e-cigarettes did not vary by acculturation level. English language participants used terms including *vape, vape pen,* and *e-cigarettes* or specific brands such as *Juul* and *Puff Bar*. Spanish language participants in the lower acculturation groups also referred to products by their English names (e.g., “fumar vapes” [smoke vapes]) or used translations of English terms (e.g., “lapicera” for “pen”).

#### Theme #4: Perceptions of the relative harm of e-cigarettes were polarized

Perceptions of the relative harm of e-cigarettes were mixed. Nearly half said e-cigarettes were the most or second most harmful among a range of nicotine and tobacco products, whereas about a quarter ranked them as the least or second least harmful. Perceived negative physical health impacts (e.g., cancer and lung disease) were associated with greater relative harm. Uncertainty or a lack of knowledge about the long-term health effects of e-cigarettes translated into perceptions of more risk/harm. Ease of access, the ability to hide/conceal e-cigarettes, and the availability of flavors were perceived as characteristics that make e-cigarettes appealing to young people, leading to repeated experimentation and therefore greater potential for addiction and harm. Conversely, perceived ability to control or moderate the nicotine content (e.g., choose e-cigarettes with lower nicotine percentages as listed on labels), amount consumed (i.e., number of hits), and frequency of use were viewed as ways of avoiding addiction and thus factored into lower harm perceptions (Table 4, 4.1). Furthermore, beliefs that vapor is less harmful than combustion, and perceptions of e-cigarettes as a cessation device, led to lower harm perceptions (Table 4, 4.2). These assessments did not differ by acculturation.

Along with health consequences, some participants described concerns around the punitive consequences of e-cigarette experimentation within their Hispanic family/community and more broadly. Strict families, a strong religious influence, and pressure to make the most of educational and career opportunities are examples that make the potential ramifications of e-cigarette use (if caught) steep. A few noted that Hispanic youth may be more adversely affected by the consequences of e-cigarette use than White youth, such as being less likely to have the resources to get out of addiction and/or to have access to treatments for withdrawal symptoms as well as being more adversely affected by the damage these inflict upon their relationships (Table 4, 4.3).

#### Theme #5: Curiosity drives e-cigarette initiation, whereas continued use is driven by peers and perceived mental health benefits

Participants explained e-cigarette initiation is driven by curiosity, influenced by: (1) flavors: participants were curious about flavors and discussed flavors with peers; (2) social norms: seeing parents or other family members, friends, or classmates vape; and (3) sensation seeking: wanting to experience a “nicotine buzz,” high, or brief change in feeling (Table 4, 5.1). Some bicultural and lower acculturation segments reported that elder relatives smoking cigarettes sparked their curiosity to try what they considered to be their generation’s product.

Continued e-cigarette use is driven by peer use and perceived mental health benefits. This included wanting to keep up with peers and maintain friendships and social pressures (e.g., looking cool) (Table 4, 5.1, 5.2). Higher acculturation and bicultural segments more often discussed experiences of direct peer pressure, whereas less acculturated segments talked more about the desire to fit in “look cool.” Participants also brought up addiction (though not always by name) as a reason for continued use. A few participants reported that continued use/experimentation with e-cigarettes was due to initiation with products that contained higher percentages of nicotine, *“hooking them”* faster. Participants frequently discussed perceived benefits to mental health (relief from anxiety or depression, particularly during the COVID-19 pandemic) as a reason for continued use (Table 4, 5.3).

#### Theme #6: Hispanic YYA face unique risk and protective factors based on their acculturation level

Bicultural and higher acculturation segments said the disconnect between generations and fear of stigma/stereotyping by family or the broader Hispanic community may make e-cigarette use more appealing because use is easy to conceal. Conversely, among lower acculturation segments, stigma and disapproval associated with e-cigarette use within the Hispanic community were deterrents.

Across segments, participants noted that family relationships influence e-cigarette use. Participants described family as central to their identity, citing familial disapproval or disappointment as a deterrent. Watching relatives struggle with addiction influenced participants’ beliefs, perceptions, and behaviors related to tobacco use. Bicultural and less acculturated segments described feeling the need to work hard and make the most of opportunities and that getting addicted to e-cigarettes would hinder their goals. These groups also mentioned “*respeto”* (respecting the sacrifices made by the older generation) more frequently (Table 4, 6.1).

## Discussion

This study points to acculturation as an important phenomenon for understanding e-cigarette experimentation, continued use, and risk and protective factors for initiation among Hispanic YYA. Findings unveiled differences related to Hispanic identity and the acculturative process, which may increase or decrease the risk of e-cigarette use. This study adds nuances to prior research examining reasons for e-cigarette use among Hispanic YYA, segmented by origin country.^8^ Our study corroborates findings around stress and curiosity as drivers of e-cigarette initiation across subgroups within the sample (e.g., age and e-cigarette use status); however, while the prior study’s authors concluded that participants shared similar cultural values, experiences, and opinions about e-cigarettes,^8^ our study suggests that differences in experiences and values around e-cigarette use differ by acculturation level (Table 4).

While more acculturated participants in our study had higher familiarity with tobacco products, e-cigarette familiarity was high across segments. Importantly, misperceptions about e-cigarette risks were also pervasive. The combination of high familiarity and misperceptions related to the relative and absolute risk of e-cigarettes may facilitate repeated e-cigarette experimentation—which we observed among more acculturated segments. These findings on e-cigarette use risk are somewhat in keeping with prior research showing that highly acculturated Hispanic YYA may be more likely to engage in riskier health behaviors (compared with lower acculturation Hispanic YYA), including combustible tobacco use.^13–17^

We also observed that familiarity with and access to tobacco products may be offset by protective factors associated with Hispanic identity, particularly among those who more strongly embrace their Hispanic culture (this refers to both the Hispanic culture more broadly and cultures related to a specific country of heritage). Our study further reinforces findings in prior literature that factors related to Hispanic identity, including *familismo* and values of hard work and self-improvement,^37,38^ may be protective against tobacco use among YYA. These factors may be useful for leveraging a strength-based approach to e-cigarette prevention interventions for Hispanic YYA. Participants across acculturation levels expressed pride in their Hispanic identity, and previous research showed identity connectedness (connection to one’s own identity as well as to the wider community) is protective against a range of negative health outcomes among the Hispanic population.^39,40^

According to prior research on acculturation and tobacco use, we might expect that a greater connection to U.S. culture would increase e-cigarette use risk, whereas a greater connection to Hispanic culture would decrease e-cigarette use risk.^16–24^ Less is known, however, about how bicultural YYA—representing a majority of Hispanic YYA—experience tension between acculturation-related risk and protective factors. Bicultural YYA in our study discussed feelings of toggling between Hispanic roots and efforts to assimilate. This included experiences of in-group discrimination related to not adhering to cultural norms or not speaking Spanish fluently and out-group discrimination related to skin color, Hispanicity, and speaking in Spanish in public. Prior research has shown that experiences of in-group discrimination, which may also include discrimination based on skin color or immigration status, can adversely affect emotional and other outcomes among Hispanic YYA and may counter the potentially protective effects of Hispanic culture.^41–43^ Although participants did not directly connect these unique risk factors with e-cigarette use, research shows that stress, internal conflict, or intrafamilial conflict related to the acculturation process are associated with increased tobacco use risk.^11,12^

Bicultural segments were similar to higher acculturation segments in their familiarity with e-cigarette products and brands and understanding of the relationship between tobacco and nicotine. However, unlike higher acculturation segments, bicultural segments had more questions and curiosity about e-cigarettes. While bicultural segments reported high deference to family and noted negative impacts of e-cigarette use on family relationships, this was not a deterrent to use like it was for lower acculturation segments. Bicultural segments considered the appeal of using a product that can be easily concealed from family members and others. Prior research has shown that Hispanic youth perceive the acculturation process to create distance between them and their parents and to negatively impact family cohesion,^44^ which is associated with e-cigarette use among minority youth, including Hispanic youth.^45^

This qualitative study suggests the association between acculturation and e-cigarette use risk may not be simply linear (i.e., more acculturation, more risk). Future studies of e-cigarette use can further unpack the unique experiences of the high percentage of bicultural U.S. Hispanic YYA.

### Limitations

These findings are not generalizable to a larger population due to the small sample size and convenience sampling. Qualitative findings do not provide quantitative probabilities, and variations in participant perspectives may exist. Additionally, this study is susceptible to self-selection, social desirability, and acquiescence biases. These findings offer novel, valuable directional insights that can be explored quantitatively with a larger sample.

## Health Equity Implications

Exploring the implications of acculturation may elucidate additional structural (e.g., discrimination) and psychosocial (e.g., acculturative stress) drivers of e-cigarette use among Hispanic YYA. Understanding unique risk/protective factors and how these vary among Hispanic YYA can inform public health education strategies to reach Hispanic YYA more comprehensively. Specifically, it would be useful to better understand the tension between community connectedness among Hispanic YYA and feelings of disconnect from Hispanic culture, especially among bicultural YYA, and how this may interplay with e-cigarette use.

Our findings show the importance of an intracategorical exploration (considering variations *within* a population or population subgroup) of Hispanic YYA e-cigarette use and may inform future research with other population subgroups facing tobacco use disparities. Future research can explore intersections between acculturation and other characteristics, including gender, national background, race, and sexual orientation. Additionally, structural-level factors (e.g., exposure to advertising and protobacco community environments) may help to explain factors associated with the risk of e-cigarette use among Hispanic YYA.

## Ethical Approval

This study was reviewed and approved by Salus IRB, no. 22095-01.

## Notes

^1^
In this article, we use the term “Hispanic” as this was the preferred term for self-identification among most of our participants. We acknowledge that the use of this term is not ubiquitous and that many identify with other terms including Latino/a/x/e. We further acknowledge that this population is highly diverse, and that many prefer to identify by their national background (e.g., country of origin such as “Salvadoran” or “Mexican”) and/or alternative racial/ethnic identities (e.g., Mestizo/a, Afro-Latino/a).

^2^
This perception may be due to labeling on products containing synthetic (“tobacco-free”) nicotine.

## Abbreviation Used

YYA: Youth and young adults
